# Effects of Ambient Particulate Matter on Human Breast Cancer: Is Xenogenesis Responsible?

**DOI:** 10.1371/journal.pone.0076609

**Published:** 2013-10-16

**Authors:** Qiang Huo, Ning Zhang, Xiaolong Wang, Liyu Jiang, Tingting Ma, Qifeng Yang

**Affiliations:** 1 Department of Breast Surgery, Qilu Hospital of Shandong University, Shandong, China; 2 School of Medicine, Shandong University, Shandong, China; 3 Key Laboratory of Experimental Teratology, Ministry of Education and Institute of Molecular Medicine and Genetics, Shandong University School of Medicine, Jinan, China; Rutgers - New Jersey Medical School, United States of America

## Abstract

**Background:**

Recently, evidence from several studies has revealed that air pollution is associated with the increased morbidity and mortality of breast cancer patients. However, to date, the underlying mechanism remains largely unclear. Considering the high prevalence of air pollution and breast cancer in China, it is necessary to understand how air pollution may affect breast cancer.

**Methods:**

We analyzed 1,832 female patients who had resided in the same cities for at least 10 years prior to their diagnosis. Variables including demographic data as well as clinical and tumor characteristics, including the patient’s age at menarche, family history of breast cancer, tumor histopathological type, tumor size, lymph node metastasis, distant metastasis, histological grade, estrogen receptor (ER) status, progesterone receptor (PR) status and human epidermal growth factor receptor 2 (HER-2) status at the time of diagnosis were analyzed.

**Results:**

Compared to patients residing in low-pollution areas, patients living in high-pollution areas demonstrated a younger age at menarche (*p*<0.001), a greater family history of breast cancer (*p* = 0.034) and more invasive cancers (*p* = 0.028) with higher tumor grades (*p* = 0.028) and estrogen receptor (ER)-positive status (*p* = 0.022). Differences in tumor grade were only found in ER-positive cases.

**Conclusions:**

Our findings and clinical data indicate that long-term air pollution exposure may contribute to the development of breast cancer by playing the role of a xenoestrogen, and also provides new insight into the association between air pollution and the morbidity and mortality of breast cancer patients. Furthermore, it is urgently necessary to study the association between air pollution and breast cancer to improve the living quality and health of females, and applicable public health strategies may need to be established or modified as soon as possible.

## Introduction

Multiple environmental factors, such as exposure to environmental pollutants and cigarette smoke, may play important roles in breast cancer development [1]. With the advance of urbanization and industrialization, the release of toxic air pollutants is prevalent in China (*National Low Carbon Development Report 2013*). Recently, evidence from several studies has revealed that air pollution is associated with the increased morbidity and mortality of breast cancer patients [2]–[4]. However, to date, the underlying mechanism remains largely unclear.

Air pollutants contain various gaseous and particulate components, and ambient particulate matter (PM) is the major air pollutant found in northern China, including the Shandong Province (information from *Environmental Protection Bureau*). PM pollutants consist of extremely small particles and liquid droplets that can contain acids, metals, organic chemicals and soil or dust particles, and PM has been shown to infiltrate human airways and be retained in the pulmonary alveoli [5]. The effects of PM exposure on human diseases constitute significant hazards to human health [6]. Thus, our study aimed to evaluate the effects of ambient air pollution on breast cancer characteristics and provide further evidence for the underlying relationship between air pollution and breast cancer using ecological data from China.

## Methods

### Study Population and Data Collection

We recruited all eligible breast cancer patients from Qilu Hospital who were diagnosed with breast cancer between 2001 and 2006. Breast cancer was confirmed by surgery, and pathological examinations were conducted for each participant. To minimize the bias caused by differences in the patients’ eating habits, lifestyle and other factors [7], [8], only long-term residents of the Shandong Province were included. To evaluate the long-term effects of air pollution on breast cancer characteristics, we only included patients who had resided in the same city for at least 10 years prior to their diagnosis.

The patients’ medical records and review information were collected, and information about the each patient’s demographic, clinical and/or pathological factors was obtained. Male patients or patients whose review information was inconsistent with their medical records were excluded. All participants have provided their written informed consent to participate in this study. The individual in this manuscript has given written informed consent (as outlined in PLOS consent form) to publish these case details. The institutional review boards of Shandong University approved all study procedures.

The class standard of ambient PM was evaluated according to the *National Standard of the People’s Republic of China, Ambient Air Quality Standard (GB3095-2000)*. In brief, the PM concentration was detected and classified according to the annual average and daily average of the PM_10_ concentration (as shown in Table 1). Information was obtained from the Environmental Protection Bureau.

**Table 1:** Concentration limit for ambient air pollutants.

Pollutant	Sampling time	Concentration limit (mg/m^3^)		
		Class 1 standard	Class 2 standard	Class 3 standard
PM_10_	Annual average	0.04	0.10	0.15
	Daily average	0.05	0.15	0.25

### Exposure Assessment and Variables

**Figure 1:**
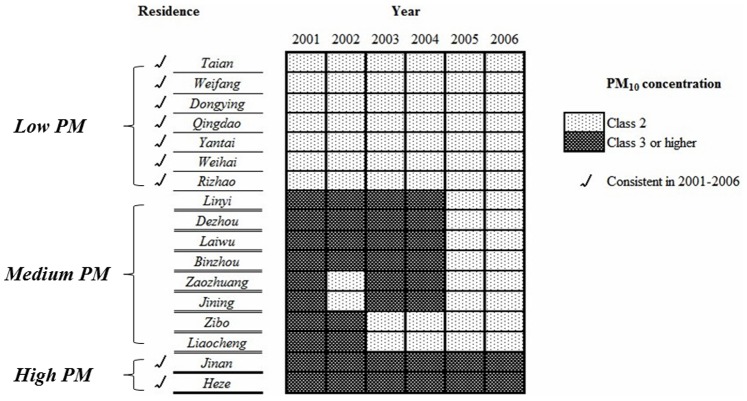
Exposure assessment and classification of participants according to residential levels of atmospheric particulate matter. Patients were classified into 3 groups according to the ambient PM_10_ class of their residence. Patients who resided in cities with Class 2 PM_10_ pollution were classified in the *Low PM* group, while the *High PM* group included cases with Class 3 or higher PM_10_ pollution. Patients living in areas with PM_10_ concentrations that varied between Class 2 and Class 3 were classified as the *Medium PM g*roup.

Patients were classified into 3 groups according to the ambient PM_10_ class of their residence, as shown in Figure 1. The dependent variables included demographic data as well as clinical and tumor characteristics, including the patient’s age at menarche, tumor histopathological type, tumor size, lymph node metastasis, distant metastasis, histological grade, estrogen receptor (ER) status, progesterone receptor (PR) status and human epidermal growth factor receptor 2 (HER-2) status at the time of diagnosis. Other variables, including the patient’s age at diagnosis, menopausal status, pregnancy status, delivery status, and breast-feeding history, were also recorded. The patient’s comorbid conditions, history of benign breast disease, receipt of neoadjuvant chemotherapy and family history of all cancer types were also recorded.

### Statistical Analyses

Multivariable logistic regression analyses of all clinical and non-clinical characteristics included the ambient PM status and cigarette-smoking status as independent variables. We excluded patients who had received neoadjuvant chemotherapy when analyzing the association between ambient PM and tumor characteristics, including histological grade, tumor size, lymph node metastasis and hormonal receptor status, as neoadjuvant chemotherapy may affect these characteristics prior to surgery or pathological examination and because the non-invasive patients did not receive neoadjuvant therapy [9], [10]. To confirm that the results reflected the long-term, rather than short-term, effects of air pollution on breast cancer patients, we further assessed our data by controlling for patient age at the time of diagnosis. We tested for all 2-way interactions; no interactions significantly affected the main results present herein, and they were therefore not included in the final model. All statistical tests were 2-sided; the odds ratios (OR) and 95% confidence intervals (CI) are presented, and *p* values less than 0.05 were considered statistically significant. All analyses were performed using the PASW Statistics 18.0 software (SPSS Inc. Chicago, Illinois, USA).

## Results

### Participant Selection and Characteristics of the Study Population

**Figure 2:**
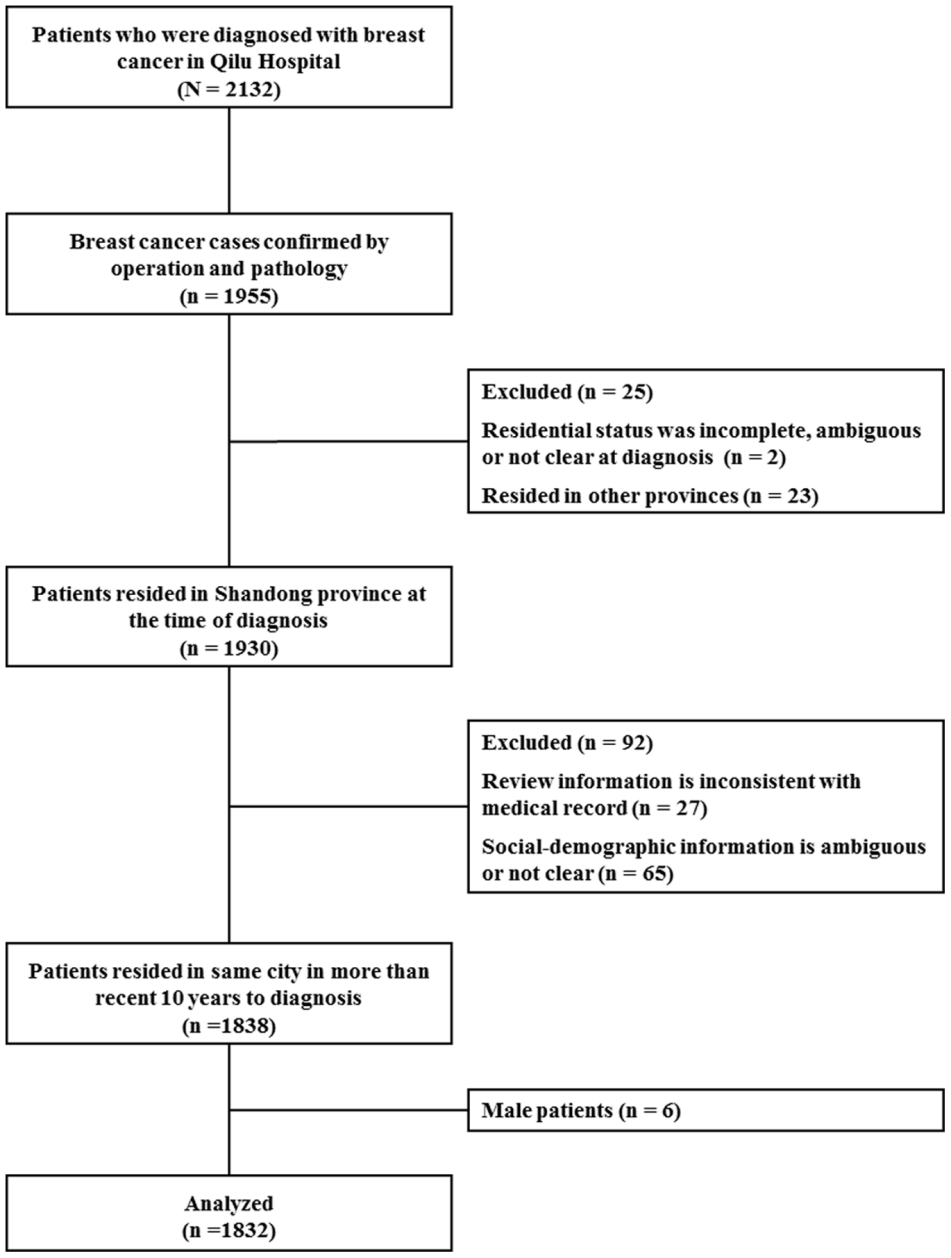
Study sample flow diagram.

We analyzed samples from 1,832 female patients who were diagnosed with invasive breast cancer (pTNM stage I-III) or ductal carcinoma *in situ* (DCIS), as shown in Figure 2. All participants were of Asian descent, and 98.85% of them were married or partnered when they were diagnosed (Table 2). The patient’s age at the time of diagnosis ranged from 22 to 87 years, and 1,036 (56.55%) cases were premenopausal females. According to the classification rules, 1,080 participants were classified in the *High PM* group, while the *Low PM* and *Medium PM* groups included 215 and 537 cases, respectively. Over half of the participants reported no smoking history, and only 3.06% (56 cases) reported a family history of breast cancer, as shown in Table 2.

**Table 2:** Demographic characteristics and key details of medical history among participants.

Characteristic	No.	Median	Minimum	Maximum
Race				
Yellow	1832			
Age at diagnosis (y)	1832	49	22	87
Age at marriage (y)	1445	24	15	42
Age at menarche (y)	1745	15	10	24
Marital status				
Married/partnered	1811			
Not married or partnered	21			
Menstrual status				
Premenopausal	1036			
Postmenopausal	752			
Pregnancy				
Never	29			
One or more	1790			
Labor				
Never	34			
One or more	1789			
Breast feeding				
Never	43			
One or more	1779			
Employment status				
Employed	709			
Self-employed	469			
Not employed	544			
Cigarette-smoking status				
Never	1531			
Past or Current	16			
Reason for visit				
Self-examination	1749			
Physical examination	71			
Comorbid conditions				
None	1139			
One or more	692			
History of breast disease or surgery				
Never	1634			
One or more	198			
History of gynecological disease or operation				
Never	1724			
One or more	108			
Family history of breast cancer				
None	1776			
One or more	56			
Air pollution				
*Low PM*	215			
*Medium PM*	537			
*High PM*	1080			

### Factors Associated with Air Pollution of Ambient PM

Our analyses identified a correlation between the PM levels and the patient’s age at menarche; a higher concentration of ambient particulates was linked with an earlier age at menarche, after adjustment for the patient’s cigarette-smoking status (OR = 0.833, 95% CI = 0.770–0.901). In addition, a larger number of participants in the *High PM* group reported a family history of breast cancer (*p* = 0.034), and similar correlations were observed between the *Medium PM* and *Low PM* groups, though these differences were not significant (as shown in Table 3).

**Table 3:** Logistic regression modeling of factors associated with atmospheric particulate matter.

Variable	*Medium PM vs. Low PM*			*High PM vs. Low PM*		
	OR	95% CI	*p* a	OR	95% CI	*p* a
Age at menarche, years	0.987	0.908–1.073	.766	0.833	0.770–0.901	**<.001**
Age at menopause, years^b^	0.952	0.885–1.025	.191	1.010	0.949–1.074	.761
Comorbid conditions						
None	Referent			Referent		
One or more	0.911	0.652–1.274	.586	1.285	0.947–1.745	.108
History of benign breast disease						
Never	Referent			Referent		
One or more	1.337	0.686–2.606	.393	1.514	0.813–2.821	.191
History of breast cancer						
Never	Referent			Referent		
One or more	1.388	0.505–3.813	.525	1.431	0.554–3.695	.459
Family history of all cancers						
Never	Referent			Referent		
One or more	0.743	0.436–1.265	.273	1.090	0.680–1.747	.720
Family history of breast cancer						
Never	Referent			Referent		
One or more	1.818	0.389–8.490	.447	4.666	1.123–19.384	**.034**
Histopathological type						
DCIS	Referent			Referent		
Invasive BC	1.422	0.843–2.399	.187	1.709	1.060–2.756	**.028**
Histologic gradec,d						
1	Referent			Referent		
2 or 3	3.105	1.323–7.287	**.009**	2.238	1.092–4.589	**.028**
Tumor sizec,d						
≤2 cm	Referent			Referent		
>2 cm	1.310	0.855–2.007	.214	0.942	0.643–1.382	.761
Lymph node metastasisc,d						
None	Referent			Referent		
One or more	1.011	0.672–1.523	.956	0.881	0.606–1.280	.506
ER status^c^						
Negative	Referent			Referent		
Positive	1.292	0.839–1.989	.245	1.576	1.069–2.323	**.022**
PR status^c^						
Negative	Referent			Referent		
Positive	1.144	0.708–1.850	.582	0.913	0.601–1.387	.670
HER-2 status^c^						
Negative	Referent			Referent		
Positive	1.123	0.609–2.071	.711	0.934	0.531–1.642	.813
Histologic grade in ER+ casesc,d						
1	Referent			Referent		
2 or 3	6.204	1.935–19.892	.**002**	3.842	1.588–9.297	**.003**
Histologic grade in ER− casesc,d						
1	Referent			Referent		
2 or 3	0.429	0.047–3.959	.456	0.619	0.072–5.350	.663

a *P* value was adjusted by cigarette-smoking status.

b After excluding cases with history of gynecological disease or operation.

c Patients without receipt of neoadjuvant chemotherapy were included in analyses.

d Only invasive cases were included in analyses.

### Ambient PM and Tumor Characteristics

We observed significant differences in the histopathological type and tumor grade of the breast cancer samples obtained from patients residing in areas of various ambient PM pollution levels, as shown in Table 3. Interestingly, 92.7% of the *High PM* cases were diagnosed with invasive breast cancer, while this proportion fell to 91.4% and 88.2% in the *Medium PM* and *Low PM* groups, respectively. A significantly increased number of invasive breast cancer cases was observed among *High PM* patients, as compared to the *Low PM* group, when the analysis was adjusted for cigarette-smoking status (OR = 1.709, 95% CI = 1.060–2.756). When the analysis focused on invasive breast cancers, we observed significant differences between cases with various levels of PM exposure; high- and medium-level exposure was associated with a higher histological tumor grade (*p* = 0.028 and *p* = 0.009, respectively). Moreover, we found that this effect of ambient PM on tumor grade was magnified in ER-positive cases (*p* = 0.003 and *p* = 0.002, respectively) but not ER-negative cases (*p* = 0.663 and *p* = 0.456, respectively).

We then reviewed the ER status, PR status and HER-2 status of the breast cancer patients in each group and found that higher levels of ambient PM correlated with ER-positive breast cancers. Furthermore, the ER status of breast cancer patients in the *High PM* group was significantly different from that of patients in the *Low PM* group (OR = 1.576, 95% CI = 1.069–2.323). No differences were observed between groups regarding PR status or HER-2 status.

## Discussion

The age at menarche is mainly determined according to the maturation of the female reproductive system and its functional coordination with the hypothalamic-pituitary-ovarian axis. As previous research has shown that estrogen plays an important role in the differentiation, maturation and function of the female reproductive system, increased estrogen exposure during puberty may contribute to the onset of menarche [11]. Considering the large sample size and the non-preferable selection of cases in the current study, hereditary factors related to these analytic variables were likely randomly distributed. We did not test the correlation of air pollution to the age of cancer diagnosis because delay in diagnosis exists and varies greatly among different cases, and the age at diagnosis of patients recorded could not reflect the time of cancer occurrence impersonally. At the same time, a greater number of participants who were exposed to high PM pollution levels reported a family history of breast cancer. As shown by medical records, the majority of these patients resided in cities with their family members, under similar air conditions. Studies have revealed that an increased breast cancer risk is associated with a greater lifetime exposure to estrogens [11], [12], and the same applies for all members of a given family. Research has also shown that the risk factors for breast cancer are not identical between these subgroups, and a long duration of estrogen exposure can have effects on the initiation and progression of ER-positive breast cancers [13]–[15]. In our analyses, the histopathological reports of patients with elevated ambient particulate exposure indicated an increased proportion of ER-positive breast cancers. When we focused on the incidence of invasive breast cancers, our analyses showed significant differences between patients with various PM exposures, with high- or medium-level exposure associated with a higher tumor histological grade. Interestingly, we found that this effect of ambient PM on tumor grade was increased in ER-positive cases but not in ER-negative cases. A recent report observed correlation of air pollution to patient survival among US females diagnosed with breast cancer [16]. We had also tried and did survival analysis and got similar results, but the response rates were too low (∼30%) to ensure that nonresponse bias does not threaten the validity of these findings (data not shown). To further confirm that these results reflected the long-term effects of air pollution on breast cancer patients and to exclude any potential short-term interference in our analyses, we further modified our analyses by controlling for patient age at the time of diagnosis, and no changes were observed in the statistical pattern of the results described.

Previous studies have presented several hypotheses to explain the correlation between air pollution and breast cancer incidence [3], [16]–[23]. For example, polycyclic aromatic hydrocarbons (PAH), a common air pollutant and a mammary carcinogen, have been shown to cause DNA damage and to contribute to the high incidence and poor prognosis of cancer patients [21]–[24]. Moreover, Lodovici et al. suggested that air pollutants may contain an environmental oxidative stressor, which could trigger redox-sensitive pathways to induce cell death and other biological processes, such as inflammation [20]. Chen et al. observed DNA damage in breast cancer cells and found significant increases in the concentration of reactive oxygen species, suggesting that aryl hydrocarbon receptor signaling was involved in this particulate-induced toxicity [19]. These findings may help to explain the observed association between air pollution and breast cancer, although additional studies are required to provide more satisfactory results.

Recently, Chen and colleagues showed that the dilution of airborne particles promoted the proliferation of ER-positive breast cancer cell lines *in vitro*. These authors confirmed that the proliferative effects induced by these airborne particles were mediated by the activation of ERα signaling, as the presence of diluted airborne particles inhibited E_2_-induced cell proliferation by approximately 75%. These findings suggest that organic solvent extracts of ambient PM demonstrate both estrogenic and anti-estrogenic effects *in vitro*, which is in line with the biological characteristics of xenoestrogens [19]. According to our analyses, we believe that long-term exposure to air pollution may contribute to breast cancer development by acting as a xenoestrogen, in addition to the effects of a hypothetical DNA-damaging agent. Although the underlying mechanisms for how air pollution impacts breast cancer development remain largely unknown and unproven, our findings highlight the need for preventive efforts to protect females from air pollution, particularly those at high risk of developing breast cancer.

### Limitations and Strengths

Several limitations of our study should be noted. First, although we selected participants from the same province to help minimize the effects of potential confounders, such as differences in lifestyle and diet, we were not able to control for differences in education level, level of physical activity or the patients’ drug intake. However, the most relevant confounder, *i.e.*, cigarette-smoking status, was introduced into our analytic models for adjustment. Second, we only considered PM_10_ data in this analysis because previous PM_2.5_ data were not accessible; these data will not be extensively monitored in China until 2016, according to the *National Standard of the People’s Republic of China, Ambient Air Quality Standard (GB3095-2000)*. Nevertheless, PM_2.5_ data were contained within the PM_10_ monitoring data because PM_10_ monitoring evaluates all PM with an aerodynamic diameter less than 10 µm. Moreover, the PM_10_ data reflect the levels of all inhalable particles that may have effects on human health through the most common route of exposure, which is absorption through the respiratory tract. Third, this research did not constitute a multicenter study, and the patients who participated in this study were recruited only from Qilu Hospital. Nonetheless, the accuracy and quality of the clinical and histopathological data are guaranteed because all cases were examined under unified techniques and standards; this level of control is important when analyzing the associations between PM pollution and histopathological characteristics, such as the histologic grade or the ER status of breast cancers. Additionally, the time spent indoors and outdoors likely varied among the participants, and this estimation was not accessible for each patient. As a consequence, we were unable to weigh the PM exposure levels with the exposure time, which may have introduced bias into our analyses. Nevertheless, it is worth noting that this situation is more likely non-differential in every group, and our sample size may have balanced out this potential bias. Meanwhile, there were 1080 patients in cities of high PM, with only 215 patients in cities of low PM. According to official statistics in 2006, we found that the population densities of areas with high or low PM were indeed very different, with 704 and 515 per square kilometers respectively, which is probably responsible for the gap of sample size in our analyses. Finally, we used city-level monitoring data to estimate the patients’ air pollution exposure, which may have resulted in misclassification of the PM exposure. Ideally, the exposure data from the closest air-monitoring point to each patient’s home or work place would reflect the most accurate estimation. Nonetheless, the data we obtained from the Environmental Protection Bureau provided a city-level evaluation of the annual and daily average of pollutants, and the monitoring point could not be classified accurately for most cases.

## Conclusions

Our findings and clinical data indicate that long-term air pollution exposure may contribute to the development of breast cancer by playing the role of a xenoestrogen. Our study also provides new insight into the association between air pollution and the morbidity and mortality of breast cancer patients. Moreover, this research highlights the need for preventive efforts to protect females from air pollution, particularly those with a high risk for developing breast cancer. Furthermore, it is urgently necessary to study the association between air pollution and breast cancer to improve the living quality and health of females, and applicable public health strategies may need to be established or modified as soon as possible.
